# Genome Sequence of the Pathogenic Fungus *Sporothrix schenckii* (ATCC 58251)

**DOI:** 10.1128/genomeA.00446-14

**Published:** 2014-05-22

**Authors:** Christina A. Cuomo, Nuri Rodriguez-Del Valle, Lizaida Perez-Sanchez, Amr Abouelleil, Jonathan Goldberg, Sarah Young, Qiandong Zeng, Bruce W. Birren

**Affiliations:** a Broad Institute of Harvard and MIT, Cambridge, Massachusetts, USA; b Medical Sciences Campus, University of Puerto Rico, San Juan, Puerto Rico

## Abstract

*Sporothrix schenckii* is a pathogenic dimorphic fungus that grows as a yeast and as mycelia. This species is the causative agent of sporotrichosis, typically a skin infection. We report the genome sequence of *S. schenckii*, which will facilitate the study of this fungus and of the *Sporothrix schenckii* group.

## GENOME ANNOUNCEMENT

*Sporothrix schenckii* is a pathogenic fungus belonging to the family *Ophiostomataceae* and is the etiologic agent of sporotrichosis (1). The most common clinical presentation of this disease is the cutaneous form with or without regional lymphatic involvement (2, 3). Sporotrichosis results from the traumatic implantation of spores into cutaneous or subcutaneous tissues. Sporotrichosis is considered to be a hazard among workers whose occupation brings them into frequent and sometimes traumatic contact with plant material or soil (4), and it is also known as “rose gardener’s disease.” Dimorphism is an important characteristic that *S. schenckii* shares with other fungal pathogens, including many species in the distantly related *Onygenales* order. *S. schenckii* can grow in the mycelium form (saprophytic form) at 25°C with hyaline, septated hyphae with small unicellular pyriform, thin-walled conidia, arranged in a daisy-like pattern at the tips of the conidiophores (5). With time, dark brown, spherical to oval, thick-walled conidia are observed attached to the hyphae. These pigmented conidia give the dematiaceous (black fungus) appearance, characteristic of *S. schenckii* colonies. *S. schenckii* can also grow in the form of spherical ovoid yeast cells (parasitic form) at 35 to 37°C (6).

To sequence the genome of *S. schenckii*, total DNA was extracted from strain ATCC 58251, a clinical isolate from Puerto Rico. Yeast cells were incubated overnight with lyticase (25 mg/ml) (Sigma Chemical Co.) prior to extraction using the Omni Prep for Fungi DNA extraction kit (G-BioSciences). The purity and concentration of the DNA were checked using a NanoDrop ND-8000 UV-Vis (Thermo, Fisher Scientific), and the integrity of the DNA was checked by agarose gel electrophoresis. For genome sequencing, we constructed two libraries, an 180-base fragment and 3-kb jumps, and sequenced both on the Illumina HiSeq 2000 platform. The 101-bp Illumina reads were assembled using ALLPATHS-LG (7) (R45374) with default parameters, from a roughly 50-fold depth of fragment reads and 100-fold depth of jumping reads.

Based on the assembly, the genome size was estimated to be 32.23 Mb, with a GC content of 55.2%. The assembly was organized in 125 contigs, which are linked by paired-end reads into 29 scaffolds. The average base is found in a scaffold with an *N*_50_ of 3.3 Mb and a contig with an *N*_50_ of 525.3 kb. A total of 8,674 protein-coding genes, 111 tRNA genes, and 20 rRNA genes were predicted by combining the output from different annotation methods as previously described (8).

*S. schenckii* is an important human-pathogenic fungus and the first member of the *S. schenckii* complex to be sequenced. This report is a major step toward the initiation of genomic studies of this complex group of fungi.

### Nucleotide sequence accession number.

The whole-genome sequence and annotation of *S. schenckii* isolate ATCC 58251 have been deposited at DDBJ/EMBL/GenBank under the accession number AWEQ01000000.
